# Induction of secondary metabolite production by UV-C radiation in *Vitis vinifera* L. Öküzgözü callus cultures

**DOI:** 10.1186/0717-6287-47-37

**Published:** 2014-09-04

**Authors:** Emine Sema Cetin

**Affiliations:** Department of Horticulture, Faculty of Agriculture and Natural Science, Bozok University, Yozgat, 66200 Turkey

**Keywords:** Callus, Secondary metabolite, UV-C treatment

## Abstract

**Background:**

The aim of the present work was to examine the role of UV-C irradiation on the production of secondary metabolites (total phenolic, total flavanols, total flavonols, catechin, ferulic acid and *trans*-resveratrol in phenolic compounds and α-, β-, γ- δ-tocopherols) in callus cultures. Studies on the effects of UV-C treatment on callus culture are seldom and generally focused on UV-B. However UV-C radiation play an important role in accumule secondary metabolites.

**Results:**

In this study, callus cultures from Öküzgözü grape cultivar were initiated from leaf petiole explants. Calli formed after 6 weeks on the medium supplemented with 0.5 mg L^-1^ benzylaminopurine (BA), 0.5 mg L^-1^ indole acetic acid (IAA) on B5 media. Callus tissues were exposed to UV-C irradiation at 10, 20 and 30 cm distances from the UV source for 5 and 10 minutes and samples were collected at hours 0, 24 and 48.

**Conclusions:**

The greatest total phenolic content (155.14 mg 100 g^-1^) was detected in calli exposed to UV-C for 5 min from 30 cm distance and sampled after 24 h. 24 h and 48 h incubation times, 30 cm and 5 min were the most appropriate combination of UV-C application in total flavanol content. Maximum total flavonol content (7.12 mg 100 g^-1^) was obtained on 0 h, 5 min and 20 cm combination. The highest (+)- catechin accumulation (8.89 mg g^-1^) was found in calli with 10 min UV-C application from 30 cm distance and sampled after 48 h. Ferulic acid content increased 6 fold in Öküzgözü callus cultures (31.37 μg g^-1^) compared to the control group. The greatest *trans*-resveratrol content (8.43 μg g^-1^) was detected in calli exposed to UV-C for 5 min from 30 cm distance and sampled after 24 h. The highest α-tocopherol concentration was found in calli exposed to UV-C for 10 min from 30 cm distance and sampled after 24 h. As a conclusion, it was showed that UV-C radiation had remarkable promoting effects on the accumulation of secondary metabolites in the calli of Öküzgözü grape cultivar.

## Background

Secondary metabolites are organic compounds synthesized by plants however they are not directly essential for photosynthesis, reproduction, respiration or other primary functions. The chemicals have extremely diverse effects. They often play an important role in the plant defense system. Some of them contribute to pollination and serve as protection from drought, salinity and UV radiation [1]. Due to their large biological activities, plant secondary metabolites have been used for centuries by humans as medicines, flavorings or recreational drugs.

Several metabolites in plants have preventive roles in diseases caused by oxidative stress [2]. The susceptibility of the human body to peroxidative damage is related to the efficiency of antioxidant defences [3]. Some secondary metabolites (e.g. polyphenols and tocopherols) have been shown to protect reactive oxygen species [4]. It is likely that they may provide unique resources for pharmaceuticals and may possess beneficial medicinal properties in humans [5]. Therefore these molecules, can be applicable to our diet and have been shown to play a possible role in the prevention of several chronic diseases [6].

The phenolic compound family is huge and comprises a complex group of compounds varying from simple phenols to highly polymerised compounds. Polyphenols have been extensively studied and are reported to possess several biological activities. Numerous studies have focused on their anti-mutagenic chemopreventive and anti-carcinogenic activities [7, 8]. Polyphenols have been shown to inhibit a wide range of enzymes such as phosphodiesterase and ATPases [7] and to enhance the production of glutathione (GSH) and GSH-related enzymes [9].

Tocopherols, collectively known as vitamin E, which are important secondary metabolites are lipidsoluble antioxidants and exclusively synthesised by photosynthetic organisms. The four known tocopherol forms (*α*, *β*, *γ* and *δ*) have the chemical structure consisting of a polar chromanol head group and a non-polar prenyl tail in common. In general, *α*-tocopherol is the major vitamin E form present in plant tissues, while *γ*-tocopherol accumulates to higher levels in most seeds [10]. Vitamin E has attracted much attention clinically because of its potential to be a very useful drug, and has been widely studied for its antiaging, anticarsinogenesis, antiatherosclerosis effects [11].

A number of strategies have been developed which aims at enhancing product formation from callus cultures. The abiotic elicitation is one of the methods for increasing the secondary metabolite production in plant cell tissue cultures. Because of the importance of the secondary metabolites, including phenolics and tocopherols on human health and nutrition, in this study, the aim was to determine the effects of UV-C irradiation as an abiotic elicitor on secondary metabolit production on *Vitis vinifera* L. cv. Öküzgözü calli.

## Results and discussion

Phenolic composition of the callus samples changed significantly according to the irradiation distance, irradiation duration and incubation time (P ≤ 0.05) and the data were given at Table 1. Total phenolic contents of the callus samples were estimated with Folin–Ciocalteu colorimetric method and UV-C irradiation seems to be an effective factor on total phenolic content. Total phenolic contents were higher on irradiated calli than control calli. The greatest total phenolic content (155.14 mg 100 g^-1^) was detected in calli exposed to UV-C for 5 min from 30 cm distance and sampled after 24 h, while the lowest values were found in control calli sampled after 48 h.

**Table 1:** **The effects of UV-C treatment on phenolic compounds in Öküzgözü calli**

Irradiation duration (min)	Irradiation distance (cm)	Harvest time (h)	Total phenolics (mg 100 g ^-1^ )	Total flavanols (mg 100 g ^-1^ )	Total flavonols (mg 100 g ^-1^ )	Catechin (μg g ^-1^ )	Ferulic acid (μg g ^-1^ )	Resveratrol (μg g ^-1^ )
Control		0	57,50^*^ l	4,69 i	0,29 i	1,82 k	4,92 kl	1,11 kl
		24	71,91 ij	4,56 i	0,29 i	1,38 k	4,38 l	1,36 k
		48	43,78 m	3,66 j	0,30 i	1,22 k	1,41 m	1,08 kl
5	10	0	76,64 h	5,05 h	2,67 e	6,92 bcd	11,07 fg	5,90 d
		24	96,25 f	5,12 h	2,46 e	7,53 b	14,42 de	2,98 gh
		48	72,99 i	5,31 gh	1,03 h	6,19 ef	9,32 ghi	3,04 fg
	20	0	73,46 hi	5,72 def	7,12 a	4,34 hi	12,41 ef	2,45ij
		24	58,45 l	5,90 d	3,32 d	3,89 i	5,97 jkl	2,06 j
		48	95,82 f	5,79 de	3,48 d	3,99 hi	8,22 hij	2,42 ij
	30	0	135,60 b	6,55 b	3,34 d	4,14 hi	22,88 c	2,52 hi
		24	155,14 a	6,86 a	5,51 b	6,70 cde	24,28 c	8,43 a
		48	128,44 cd	7,03 a	3,41 d	7,30 bc	31,37 a	3,46 f
10	10	0	66,50 k	5,12 h	2,48 e	6,16 ef	10,27 fgh	2,00 j
		24	69,13 jk	5,26 gh	1,03 h	4,61 h	10,06 fgh	2,53 hi
		48	87,39 g	5,06 h	1,62 g	6,39 de	7,07 ijk	2,47 ij
	20	0	73,53 hi	5,19 h	3,39 d	2,60 j	23,12 c	2,95 gh
		24	85,16 g	5,29 gh	3,12d	2,64 j	16,75 d	5,92 d
		48	113,02 e	5,21 h	5,55 b	4,22 hi	30,81 ab	7,48 b
	30	0	126,00 d	6,17 c	2,12 f	5,58 fg	15,61 d	6,93 c
		24	113,02 e	5,60 ef	3,17 d	5,26 g	28,44 b	4,72 e
		48	130,40 c	5,50 fg	2,62 e	8,89 a	29,10 ab	2,44 ij

^*^Differences between means indicated by the same letters are not statistically significant ( *p* ≤ 0.05).

Total flavanol contents were also positively affected by the UV-C irradiation. Provided that 24 h and 48 h incubation times, 30 cm and 5 min was the most appropriate combination of UV-C application in terms of total flavanol content. The lowest total flavanol contents as well as total phenolic contents were obtained from the control calli.

Total flavonol contents were significantly higher at UV treatments when compared to control.

Maximum total flavonol content (7.12 mg 100 g^-1^) was obtained when incubation time, irradiation duration and irradiation distance were set to be 0 h, 5 min and 20 cm above the calli, respectively. Present results show that irradiation with UV-C light enhances phenolic production by callus cultures of Öküzgözü grape cultivar.

In this study, not only the total phenolic, flavanol and flavonol contents were determined by spectrophotometric methods, but also some individual phenolics (only detectable) including (+)-catechin, ferulic acid and *trans*-resveratrol were defined by HPLC. HPLC method for analyzing phenolics in the samples has some advantages such as easy and fast procedure for the preparation of the samples, possibility of quantification of diverse phenolics, the precision, accuracy and low detection limits d which enable its application to both grape and wine [12].

(+)- Catechin is the most abundant flavanoid in grape seed, skin and wine [13, 14]. (+)- catechin has some beneficial effects on human health. It prevents LDL oxidation and has antifungal effects [15, 16]. For these reasons, (+)- catechin accumulation regulated with the UV-C treatment was determined in this study. The irradiation duration and distance, as well as the incubation time appeared to be the major factors influencing the accumulation of (+)- catechin. The highest (+)- catechin accumulation (8.89 mg g^-1^) was found in calli with 10 min UV-C application from 30 cm distance and sampled after 48 h. On the other hand, the lowest values were detected in control calli.

Stimulation of the synthesis of proanthocyanidins by UV radiation, including catechin, a monomer of proanthocyanidin, might be explained by the fact that they acts as an absorbant in the UV region of the spectrum and therefore capable of protecting plant cells from the harmful effects of UV [17].

Ferulic acid, like many phenols, has antioxidant activity [18]. Ferulic acid was the phenolic compound which best responded to UV-C irradiation among all phenolics. Ferulic acid content increased 6 fold in Öküzgözü callus cultures (31.37 μg g^-1^) compared to the control group.

Present results confirm that the elicitation with UV stimulates production of some phenolics in grape callus cultures. Chappel and Hahlbrock [19] claimed that UV light is required for flavonoid production, and enhances phenylalanine ammonia lyase (PAL) and chalcone synthase (CHS) mRNAs in parsley cell cultures. Similarly, Antognoni et al. [20] studying on *Passiflora* callus cultures reported that callus cultures treated with UV showed a higher flavonoid production compared to untreated calluses. Their results showed that the secondary metabolite biosynthetic capacity of *Passiflora* tissue cultures can be enhanced by UV irriadition.

Resveratrol (3, 4, 5-trihydroxystilbene) is a phytoalexin, which belongs to stilbene family and has important roles in the protection against fungal infections [21]. Resveratrol synthesis mainly takes place in grapevine. Recently, the attention on resveratrol significantly increased due to its importance on human health as an antioxidant, having protectory roles against coronary diseases [22] and cancer [23] and also due to its great potential to be used in medicinal, pharmaceutical, food, agriculture and cosmetic industies. In this respect, the production of this compound is important and callus cultures can be used as a source.

In this study, the production of the *trans*-resveratrol in callus cultures of Öküzgözü grape cultivar was changed according to the UV radiation duration, distance from callus and sampling time. The greatest *trans*-resveratrol content (8.43 μg g^-1^) was detected in calli exposed to UV-C for 5 min from 30 cm distance and sampled after 24 h and in this application *trans*-resveratrol was 8 fold higher than those of control calli. Our results confirmed the findings of Keskin and Kunter [24] which stated that Öküzgözü calli exposed to UV-C produced higher amounts of *trans*-resveratrol when compared to untreated calli.

Cantos et al. [25] also reported that the production of the *trans-*resveratrol in calus culture of grape was changed depending on the duration and distance of UV-C radiation. They found the maximum *trans-*resveratrol yields when UV-C irradiation duration and distance were set to be 30 s and 40 cm above the grapes, respectively. Keskin and Kunter [26] studied the effects of UV-C radiation on *trans-*resveratrol content of Ercis grape calli treated with two different irriation duration (10 and 15 min) and found that the highest *trans-*resveratrol concentration was at 48 hours of 12 days-old callus cultures irradiated for 10 min. Similarly, to induce *trans-*resveratrol, calluses were exposed to the UV-C irradiation and significant quantities of *trans-*resveratrol was produced by calluses of *Arachis hypogaea* upon UV irradiation [27].

In this research, the effects of UV-C treatments on tocopherol accumulation was also examined. UV treatments on plant calli generally focuses on phenolic compounds. With our best knowledge, there is no research on the accumulation of tocopherols in plant calli. This paper mentions the use of UV treatment to enhance tocopherols in calli for the first time.

Tocopherol composition of the callus samples changed significantly according to the irradiation distance, irradiation duration and incubation time (P ≤ 0.05) and data were given Table 2. α, β and γ-tocopherols were found at different concentrations depending on the treatments, while δ-tocopherol was not detected in calli of Öküzgözü. Within the calli collected at 0 h, control group had the most abundant α-tocopherol content compared to the UV-C treated group, independent of the duration and distance of treatment. For the calli sampled after 24 h and 48 h, it was shown that 5 min UV-C treatment did not enhance α-tocopherol content in calli and these calli had lower α- tocopherol content than the control calli. However, when the UV-C treatment distance was increased, 10 min applications increased the α-tocopherol concantration compared to control calli sampled after 24 h and 48 h. Our data indicated that the highest α-tocopherol concentration was found in calli exposed to UV-C for 10 min from 30 cm distance and sampled after 24 h. On the other hand β and γ- tocopherols were higher on control group than irradiated calli. In summary, it can be concluded that, there is a positive effect of UV irradiation on the content of α-tocopherol depending on the irradiation duration, distance from samples and incubation time for grape calli, while UV-C treatments had no positive effect on β and γ- tocopherol accumulation in calli. The analyses conducted in the present work revealed that these conditions are the optimum conditions which stimulate the biochemical pathways leading to α-tocopherol accumulation.

**Table 2:** **The effects of UV-C treatment on tocopherols in Öküzgözü calli**

Irradiation duration (min)	Irradiation distance (cm)	Harvest time (h)	Tocopherols (μg 100 g ^-1^ )		
			α tocopherol	β tocopherol	γ tocopherol
Control		0	77,68^*^ g	13,32 c	13,18 c
		24	133,12 d	16,38 a	18,67 a
		48	106,56 f	15,24 b	14,58 b
5	10	0	67,30hi	5,14 ef	5,19 e
		24	51,45 k	1,12 i	1,08 h
		48	43,42 l	2,12 gh	4,25 f
	20	0	58,22 j	2,42 hi	2,96 f
		24	43,32 l	3,45 gh	3,45f
		48	43,00 l	2,17 hi	5,42 e
	30	0	62,47 ij	5,27 ef	9,85 d
		24	65,17 hi	2,14 hi	5,31 e
		48	56,84 j	1,16 i	1,00 h
10	10	0	40, 48 l	2,18 hi	3,27 f
		24	82,38 g	3,36 gh	3,86 f
		48	70,42 h	3,24 gh	2,36 g
	20	0	64,75 hi	3,97 fg	5,42 e
		24	190,82 b	3,42 gh	4,09 f
		48	126,84 e	3,32 gh	1,12 h
	30	0	51, 38 k	3,16 gh	4,39 f
		24	261,47 a	10,48 d	3,42 f
		48	155,72 c	6,37 e	2,89 g

^*^Differences between means indicated by the same letters are not statistically significant ( *p* ≤ 0.05).

Irradiation by all UV wavelengths damages a number of plant processes [28, 29]. The damages of UV irradiation activate some mechanisms involving endogenous sensitizers and the generation of active oxygen species [30]. Primary radicals formed as a result of UV irradiation lead to the formation of lipid radicals, which react with oxygen to produce lipid peroxy radicals [31]. *In vitro* experiments have demonstrated that α-tocopherol has the ability to terminate chain reactions of polyunsaturated fatty acid free radicals generated by lipid oxidation and therefore, α-tocopherol acts as an efficient chain-breaking antioxidant [32].

The effects of UV irradiation on tocopherol contents change depending on the genotypes. Kacharava et al. [33] informed that kidney bean varieties and white beet responded to UV irradiation with increased levels of tocopherol, although the responses of red beet was not similar. Tocopherols decreased in red beets exposed to UV.

Environmental stresses including light, temperature, and oxygen affect and alter α-tocopherol content by inducing oxidative damage [34–36]. Kozak et al. [31] found that UV-B + UV-C irradiation enhanced α-tocopherol contents two times more in surface layers of soybean cotyledons than control and UV-B irridiated materials. They explained this situation as UV-B and UV-C irradiation affect different mechanisms at the cellular level and UV-C irradiation triggers molecular signals in common with more than one metabolic pathway.

## Conclusions

In this study, the effects of UV-C irradiation on phenolic compounds and tocopherols were investigated. The results of the study showed that UV-C radiation had remarkable promoting effects on the accumulation of phenolics in the calli of Öküzgözü grape cultivar. However, further studies investigating various strategies on secondary metabolite, especially tocopherol production, should also be carried out.

## Methods

Callus tissues obtained from leaf petioles of Öküzgözü grape cultivar were used as plant material. Petioles were surface sterilised with bleach (15%) for 15 min and rinsed. Petioles were then cut into 1 cm pieces and placed onto solid B5 [37] culture medium with 30 g L^-1^ sucrose and 8 g L^-1^ bacto agar supplemented with 0.5 g L^-1^ benzylaminopurine (BA), 0.5 g L^-1^ indole acetic acid (IAA). The pH was adjusted to 5.75. All cultures were incubated in a growth cabine under dark at 25 ± 1°C. Induced calli were subcultured on the same media in order to maintain sufficient stock cultures.

### Exposure to UV-C radiation

For investigation of the effect of UV-C irradiation on secondary metabolite production, UV-C lamp (Philips TUV-6 W, 254 nm wavelength, used for experimental purposes) was used as elicitor. For the elicitor applications, calli were transferred to fresh media at the same culture conditions and all experiments were carried out in 150 mL magenta boxes containing 50 mL medium and left for growth for 12 days. The UV light was applied from distances of 10, 20 and 30 cm for 5 (aprox. 25.2-57.6 kJ cm^-2^) and 10 minutes (aprox. 50.4-115.2 kJ cm^-2^) onto the 12-day-old cultures by the removal of the cover of magenta boxes inside a sterile cabin. Controls were 12-day-old cultures untreated with UV-C irradiation. Callus samples were taken at three different periods of time (0, 24, and 48 h) from initial calli for both the treatment and control group. Callus samples were crushed in liquid nitrogen and stored at -20°C until the analyses.

### Extraction for phenolic compounds

Phenolic extraction was done according to the modified procedure of Kiselev et al. [38]. Powdered callus culture sample (2 g) was extracted with 96% EtOH overnight at a temperature of 45°C. After, homogenate was centrifuged at 4000 rpm for 5 min. The supernetant was evaporated under vacuum in a rotary evaporator at 45°C to dryness. The dry residue was dissolved in 2 mL of MeOH. After filtering through a 0.22 μm Millipore filter, filtrates were directly used for spectrophotometric and HPLC analyses.

### Determination of phenolic compounds

Total phenolic, total flavanols and total flavonol contents of the samples were determined spectrophotometrically using a PG Instruments T70 Plus Dual Beam Spectrophotometer (Arlington, MA, USA). Total phenolic contents were determined according to the Folin-Ciocalteu colorimetric method [39], calibrating against gallic acid standards and expressing the results as mg gallic acid equivalents (GAE) (mg 100 g^-1^). Total flavanol contents were determined according to the Arnous et al. [40], calibrating against catechin standards and expressing the results as mg catechin equivalents (CE) (mg 100 g^-1^). Total flavonol contents were determined according to Dai et al. [41], calibrating against rutin standards and expressing the results as mg rutin equivalents (RE) (mg 100 g^-1^). Data presented are average of the three measurements.

HPLC analyses were carried out as previously described by Caponio et al. [42]. The HPLC system (Shimadzu Corp., Kyoto, Japan) was equipped with a pump (LC 10 AD), auto-sampler (SIL 10 AD), column oven (CTO 10A) and diode-array UV/VIS detector (DAD-λmax = 278). The separation was executed on a Agilent Eclipse XB C-18 (5 μm, 4.6 × 250 mm, Wallborn,Germany). The mobile phase was composed of acetic acid (2%) and methanol with the gradient elution system at a flow rate of 0.8 mL min^-1^. For gradient elution, mobile phase A contained 3% acetic acid in water; solvent B contained methanol. The following gradient was used: 0–3 min, from 100% A to 95% A, 5% B; 3–20 min, from 95% A, 5% B to 80% A, 20% B; 20–30 min, from 80% A, 20% B to 75% A, 25% B; 30–40 min, from 75% A, 25% B to 70% A, 30% B; 40–50 min 70% A, 30% B to 60% A, 40% B; 50–55 min, 60% A, 40% B to 50% A, 50% B; 55–65 min, 50% A, 50% B to 100% B. The injection volume was 20 μL. Samples, standard solutions and mobile phases were filtered by a 0.45 μm pore size membrane filter (Millipore Co. Bedford, MA). The detection UV wavelength was set at 280 nm. The column temperature was set at 30°C. The compounds were quantified using Shimadzu CLASS-VP software. Catechin, ferulic acid and *trans*-resveratrol contents were determined on HPLC expressing the results as μg g^-1^. Data presented are average of the three measurements.

### Determination of tocopherols

The extraction of tocopherols (α, β, γ and δ-tocopherol) were carried out as previously described by Caretto et al. [43]. Briefly, the method consisted of an alkaline hydrolysis (potassium hydroxide 60%) followed by extraction with *n*-hexane-ethyl acetate (9:1). Chromatography separation was performed by using a Beckman HPLC Analytical System. Luna Silica (250 × 4.6 mm) 5 μ column was used with Heptane:tetrahydrofuran (95:5) as the mobile phase. RF-10AXL Floresan dedector was used to determine tocopherols. The tocopherol content was calculated as μg 100 g^-1^ FCW (Fresh Cell Weight). Each experiment was carried out as three replicates.

### Statistical analysis

Data were subjected to analysis of variance with mean separation by Duncan’s multiple range test. Differences were considered statistically significant at the *p* ≤ 0.05 levels. Statistical analysis was performed using SPSS 16.0 for Windows.
